# CT Air Trapping Is Independently Associated with Lung Function Reduction over Time

**DOI:** 10.1371/journal.pone.0061783

**Published:** 2013-04-16

**Authors:** Onno M. Mets, Pim A. de Jong, Bram van Ginneken, Cas L. J. J. Kruitwagen, Mathias Prokop, Matthijs Oudkerk, Jan-Willem J. Lammers, Pieter Zanen

**Affiliations:** 1 Radiology, University Medical Center Utrecht, Utrecht, The Netherlands; 2 Diagnostic Image Analysis Group, Radboud University Nijmegen Medical Centre, Nijmegen, The Netherlands; 3 Julius Center for Health Science and Primary Care, University Medical Center Utrecht, Utrecht, The Netherlands; 4 Radiology, Radboud University Nijmegen Medical Centre, Nijmegen, The Netherlands; 5 Radiology, University Medical Center Groningen, Groningen, The Netherlands; 6 Division of Heart and Lungs, University Medical Center Utrecht, Utrecht, The Netherlands; University of Navarra, Spain

## Abstract

**Purpose:**

We aimed to study the association between lung function decline and quantitative computed tomography (CT) air trapping.

**Materials and Methods:**

Current and former heavy smokers in a lung cancer screening trial underwent volumetric low-dose CT in inspiration and expiration. Spirometry was obtained at baseline and after 3 years. The expiratory to inspiratory ratio of mean lung density (E/I-ratio_MLD_) was used to quantify air trapping. CT emphysema was defined as voxels in inspiratory CT below −950 Hounsfield Unit. Linear mixed modeling was used to determine the association between CT air trapping and lung function.

**Results:**

We included 985 subjects with a mean age of 61.3 years. Independent of CT emphysema, CT air trapping was significantly associated with a reduction in forced expiratory volume in one second (FEV_1_) and the ratio of FEV_1_ over the forced vital capacity (FEV_1_/FVC); FEV_1_ declines with 33 mL per percent increase in CT air trapping, while FEV_1_/FVC declines 0.58% per percent increase (both p<0.001). CT air trapping further elicits accelerated loss of FEV_1_/FVC (additional 0.24% reduction per percent increase; p = 0.014).

**Conclusion:**

In a lung cancer screening cohort, quantitatively assessed air trapping on low-dose CT is independently associated with reduced lung function and accelerated decline of FEV_1_/FVC.

## Introduction

Chronic obstructive pulmonary disease (COPD) causes chronic morbidity and mortality, and is expected to be the third leading cause of death in 2020, with around 8 million deaths annually [1]; [2]. COPD is characterized by progressive airflow limitation due to parenchymal destruction (i.e. emphysema) and/or small airways remodeling, and is primarily caused by exposure to tobacco smoke [3]. It has been reported that not all smokers are susceptible to the harmful effects of tobacco, and only a subgroup has a decline in lung function large enough to develop COPD [4]. Since smoking cessation is crucial in managing this disease [5], it would be advantageous to estimate the rate of decline in heavily smoking subjects without or with early stage COPD. Given the high expectations of lung cancer screening [6], CT may gain a role in early identification of such subjects [7].

Both pulmonary emphysema and air trapping can nowadays be quantified in vivo using computed tomography (CT), but the relationship between quantitative CT measurements and lung function decline over time received little attention. It has been reported that visual [8] and quantitative CT measures of emphysema [9]–[11] and hyperinflation [12] are associated with loss of lung function over time, and may thus be used to identify subjects at a higher risk to develop COPD. Given that airflow obstruction in COPD starts in the small airways before the onset of emphysematous destruction [13], air trapping -which is thought to reflect small airways disease- might show a strong and more important association with lung function decline in early stages of the disease, independent of emphysema. However, to date no studies have investigated the relationship between lung function decline and expiratory CT data. Therefore, the objective of this study is to assess the association between lung function decline and quantitative CT measures of air trapping in a cohort of male heavy smokers in a lung cancer screening setting.

## Materials and Methods

### Ethics statement

This study was performed as part of the population-based Dutch Belgian Lung Cancer Screening Trial (NELSON-trial) [14], which was approved by the Dutch Ministry of Health and by the ethical review board of the University Medical Center Utrecht. In our center, expiratory CT was added to the screening protocol in July 2007 to study COPD. This addition was separately approved by the ethical review board of the University Medical Center Utrecht. Written informed consent was obtained from each individual participating in the screening trial.

### Study subjects

Participants in the screening trial are current or former heavy smokers who have smoked at least 16.5 packyears and were physically fit enough to undergo potential surgery [14]. For this study, we included all subjects from our center with a lung function test in the first round of the screening and a paired inspiratory and expiratory CT, processed for quantitative CT estimates of emphysema and air trapping (N = 985). Multiple pulmonary function tests were available for 442 of these 985 subjects, spanning an observation time of around three years.

### Pulmonary Function Testing

Prebronchodilator spirometry was performed using ZAN equipment (ZAN Messgeräte GmbH, Oberthulba, Germany), according to American Thoracic Society and European Respiratory Society guidelines [15]. Spirometry was obtained between 2004 and 2008, and provided forced expiratory volume in one second (FEV_1_), forced vital capacity (FVC) and the FEV_1_/FCV ratio.

### CT scanning and quantitative analysis

All subjects received low-dose volumetric CT in inspiration and at end-expiration after standardized breathing instructions. CT imaging was obtained between July 2007 and September 2008. The images were acquired with 16×0.75 mm collimation (Brilliance 16P; Philips Medical Systems, Cleveland OH, USA). Settings were adjusted to body weight of the patient: 120 kVp (≤80 kg) or 140 kVp (>80 kg) both at 30 mAs for inspiratory CT, and 90 kVp (≤80 kg) or 120 kVp (>80 kg) both at 20 mAs for expiratory CT. A scan pair yielded an estimated effective dose of 1.2–2.0 millisievert (mSv), of which 0.3–0.65 mSv is accounted for by the expiration scan. Images with slice thickness of 1.0 mm at 0.7 mm increment were reconstructed from lung bases to lung apices using a smooth reconstruction kernel (B-filter; Philips).

The lungs were automatically segmented using dedicated software [16], and a noise reduction filter was applied to decrease the influence of noise on the quantitative measurements [17]. The density of each voxel in the segmented lung volume was assessed and distributed in an attenuation histogram. From these histograms the quantitative CT measures were calculated. CT air trapping was defined as the expiratory to inspiratory ratio of mean lung density; E/I-ratio_MLD_ [18]; [19]. CT emphysema was defined as the percentage of voxels in inspiratory CT with an attenuation below −950 Hounsfield Unit (HU); IN_−950_ [20], [21].

### Statistical analysis

It has been shown that lung function decline is linear over a three year period [22]. FEV_1_ and FEV_1_/FVC were therefore analyzed with a random slope, random intercept linear mixed model. Observation time was chosen as a random parameter, while all other parameters were fixed. We used an unstructured covariance matrix. Quantitative CT air trapping, CT emphysema, age, height, smoking status, packyears smoked and observation time were inserted into the model. We inserted the interaction between smoking status and observation time to test for differences in decline between current- and former smokers. We also inserted the interactions between CT air trapping and observation time to test whether differences in decline were dependent on the extent of CT air trapping.

Observation time and quantitative CT measures are expressed as median with interquartile range (IQR), all other data are presented as mean ± standard deviation (SD). All analyses were performed with SPSS Version 19.0 for Windows (SPSS, Chicago, Illinois, USA). A p-value below 0.05 was considered significant.

## Results

### Study population

The total study population consisted of 985 subjects (99.1% males) with an age of 61.3±5.5 years. Current and former smokers were about equally present. Average FEV_1_ at baseline was 3.28±0.71 L (96.5±18% of predicted value), and average FEV_1_/FVC at baseline was 71.6±9.2%. Study population characteristics are summarized in Table 1.

**Table 1:** Characteristics of the study population.

	N = 985
Male, n (%)	976 (99.1)
Age [year], mean ± SD	61.3±5.5
Length [cm], mean ± SD	178±7
Packyears [year], mean ± SD	40.6±17.5
Smoking status	
Current smoker, n (%)	528 (53.6)
Former smoker, n (%)	457 (46.4)
FEV_1_ [L], mean ± SD	3.28±0.71
FEV_1_ [%predicted], mean ± SD	96.5±18.0
FEV_1_/FVC [%], mean ± SD	71.6±9.2
Airflow obstruction^a^	
No COPD	624 (63.4)
GOLD 1	235 (23.9)
GOLD 2	107 (10.9)
GOLD 3	19 (1.9)
CT Emphysema, IN_−950_ [%], median (IQR)	0.66 (0.32–1.38)
CT Air trapping, E/I-ratio_MLD_, median (IQR)	0.84 (0.80–0.88)
Number of PFT	
One PFT, n(%)	543 (55.1)
Two PFT, n(%)	369 (37.5)
Three PFT, n(%)	68 (6.9)
Four PFT, n(%)	5 (0.5)
Observation time [year], median (IQR)^b^	2.9 (2.9–3.0)

a Airflow obstruction defined as FEV_1_/FVC<0.70, and classified as GOLD 1 (FEV_1_≥80%), GOLD 2 (50%≤FEV_1_<80%) and GOLD 3 (FEV_1_<50%); ^b^follow-up time in years between multiple visits;

*FEV_1_/FVC* ratio of FEV_1_ over forced vital capacity; *FEV_1_* forced expiratory volume in the first second; *IN_−950_* percentage of voxels in inspiratory CT with an attenuation below −950 Hounsfield Unit; *Perc_15_* 15^th^ percentile of the attenuation distribution curve; *E/I-ratio_MLD_* expiratory to inspiratory ratio of mean lung density; *PFT* pulmonary function test.

### Association with lung function

More extensive CT air trapping was significantly associated with a reduction in FEV_1_ (p<0.001). For each 1% increase in CT air trapping the FEV_1_ is lowered by 33 ml; roughly the annual decline in healthy male subjects. The estimated effect sizes of the variables on FEV_1_ are shown in Table 2.

**Table 2:** Results of linear mixed model analysis–change in lung function parameter per unit change in covariable.

Estimated effects of covariables on FEV_1_ (mL)				
Variable	Change	FEV_1_ difference	95%CI	p-value
log CT emphysema^a^	Plus 1 Unit	−31.1	−53.7–−8.53	0.007
CT air trapping^b^	Plus 1%	−33.4	−39.1–−27.7	<0.001
Smoking status	Current smoker	−112.8	−183.7–−41.9	0.002
Packyears	Plus 1 year	−3.9	−5.8–−1.9	<0.001
Age in years	Plus 1 year	−32.9	−39.6–−26.3	<0.001
Length in cm	Plus 1 cm	38.9	33.7–44.2	<0.001
Observation time	Plus 1 year	−56.7	−70.0–−43.5	<0.001
Current smoker * Observation time	Plus current smoker * 1 year	−26.7	−44.7–−8.7	0.004

a CT emphysema defined as the log transformed percentage of voxels below −950 Hounsfield Units (IN_−950_); ^b^CT air trapping defined as the expiratory to inspiratory ratio of mean lung density (E/I-ratio_MLD_); *FEV_1_/FVC* ratio of FEV_1_ over forced vital capacity; *FEV_1_* forced expiratory volume in the first second;

The effect of the covariable on FEV_1_ can be determined by calculating the product of the coefficient (ie. FEV_1_ difference) times the increase in covariable (ie. Change). For example, the maximum loss of FEV_1_ due to CT emphysema in our population is about 45 mL (ie. log[range_CT Emphysema_]*coefficient) compared to 1276 mL due to CT air trapping (ie. range_CT Air trapping_*coefficient).

**Figure 1:**
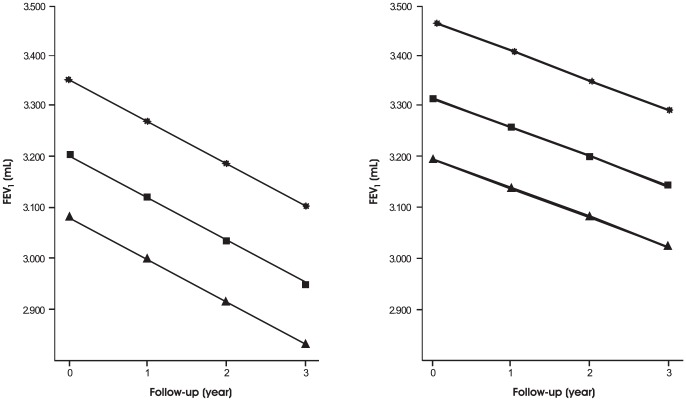
The effect of increase in CT air trapping extent on FEV_1_. The effect of increasing extent of CT air trapping (25^th^ percentile, stars; 50^th^ percentile, squares; 75^th^ percentile, triangles) on FEV_1_ is shown in a current (left panel) and former smoker (right panel) with fixed values for age/length/packyears (mean of the study population) and CT emphysema (median of the study population). It is seen that more extensive CT air trapping leads to a reduction in FEV_1_.

Increase in height predictably leads to a higher FEV_1_, while increases in CT air trapping, CT emphysema, age, packyears smoked, observation time and being a current smoker all reduce the FEV_1_. Moreover, current smokers show an accelerated decline over time, compared to non-smokers (p = 0.004). CT air trapping was not significantly associated with an additional accelerated decline in FEV_1_. The effect of increase in CT air trapping on FEV_1_ is further illustrated in Figure 1.

**Figure 2:**
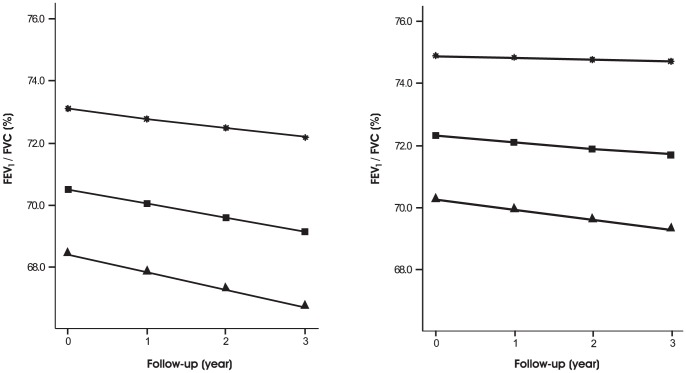
The effect of increase in CT air trapping extent on FEV_1_/FVC. The effect of increasing extent of CT air trapping (25^th^ percentile, star; 50^th^ percentile, square; 75^th^ percentile, triangle) on FEV_1_/FVC is shown in a current (left panel) and former smoker (right panel) with fixed values for age/length/packyears (mean of the study population) and CT emphysema (median of the study population). It is seen that more extensive CT air trapping leads to a reduction in FEV_1_/FVC, and the diverging course illustrates the association between CT air trapping and accelerated decline.

CT air trapping was also significantly associated with a reduction in FEV_1_/FVC (p<0.001). For each 1% increase in CT air trapping the FEV_1_/FVC is lowered by 0.58%, roughly three times the annual decline of 0.18% in healthy male subjects. The estimated effect sizes of the variables on FEV_1_/FVC are shown in Table 3. Increase in CT air trapping, CT emphysema, packyears smoked, observation time and being a current smoker all reduce the FEV_1_/FVC. Additionally, CT air trapping not only lowers the FEV_1_/FVC, but also elicits an accelerated loss (p = 0.014). Smoking status was not significantly associated with an accelerated decline in FEV_1_/FVC. Our results show that when more extensive air trapping is present, the steeper this lung function parameter will decline. When CT air trapping worsens from the 25^th^ percentile (0.80) to the 75^th^ percentile (0.88) the FEV_1_/FVC decline increases with an extra 0.24% per year, which is substantial given the normal annual decline of 0.18%. The effect of increase in CT air trapping on FEV_1_/FVC is further illustrated in Figure 2.

**Table 3:** Results of linear mixed model analysis–change in lung function parameter per unit change in covariable.

Estimated effects of covariables on FEV_1_/FVC (%)				
Variable	Change	FEV_1_/FVC difference	95%CI	p-value
log CT emphysema^a^	Plus 1 Unit	−2.68	−3.00–−2.36	<0.001
CT air trapping^b^	Plus 1%	−0.58	−0.65–−0.50	<0.001
Smoking status	Current	−1.82	−2.70–−0.94	<0.001
Packyears	Plus 1 year	−0.04	−0.06–−0.01	0.004
Observation time	Plus 1 year	+2.50	0.35–4.65	0.02
CT air trapping * Observation time	Plus 1% * 1 year	−0.03	−0.06–−0.01	0.01

a CT emphysema defined as the log transformed percentage of voxels below −950 Hounsfield Units; ^b^CT air trapping defined as the expiratory to inspiratory ratio of mean lung density; *FEV_1_/FVC* ratio of FEV_1_ over forced vital capacity; *FEV_1_* forced expiratory volume in the first second;

The effect of the covariable on FEV_1_/FVC can be determined by calculating the product of the coefficient (ie. FEV_1_/FVC difference) times the increase in covariable (ie. Change). For example, the maximum loss of FEV_1_/FVC due to CT emphysema in our population is about 3.9% (ie. log[range_CT Emphysema_]*coefficient) compared to 22.2% due to CT air trapping (ie. range_CT Air trapping_*coefficient).

## Discussion

This study on CT air trapping and lung function decline in heavily smoking male lung cancer screening participants showed that more extensive CT air trapping is associated with a substantial accelerated decline of FEV_1_/FVC, but not of FEV_1_. CT air trapping is further associated with reduced FEV1/FVC and FEV1, independent from CT emphysema.

No previous studies assessed the association between expiratory CT measures and lung function over time, and only a few papers assessed this association for inspiratory CT measures. These studies mostly found that increasing emphysema was associated with accelerated decline in lung function. Remy-Jardin et al. [8], in a visual assessment study in 111 volunteers, found that persistent current smokers with emphysema showed more rapid lung function decline than subjects without CT abnormalities. This has been confirmed by several quantitative studies [9]–[11] in which the extent of CT emphysema was related to a larger reduction in lung function over time. Contrarily, Yuan et al. [12] were unable to find an association between quantitative CT emphysema and lung function decline in 143 subjects, but they did report a weak association between hyperinflation on inspiratory CT (defined as the percentage of total lung volume that had an inflation value above the maximal predicted inflation value) and accelerated annual decline of FEV_1_.

The present study is compatible to the available literature. Our observation that CT air trapping, and not CT emphysema, elicits a steeper FEV_1_/FVC decline over time is in line with the recent evidence that COPD starts within the small airways and precedes emphysematous parenchymal destruction [13]. Also, as the subjects in the present study had mainly absent or mild obstruction, our findings are compatible with the idea that small airways disease leads to air trapping before emphysema develops. Our findings may thus suggest that small airways dysfunction is more important than emphysema for lung function decline in early disease. Nevertheless, it is important to realize that although our findings are compatible with literature our study does not proof that small airways disease develops earlier than emphysema. Also, although presumed, there is no definitive proof that CT air trapping measures pure small airways disease due to the lack of a true gold standard.

We further showed the significant relationship between a lower lung function and both CT emphysema and CT air trapping extent. However, the contribution of CT emphysema to the lung function reduction in our study population was limited compared to the effect of CT air trapping; this is illustrated by the calculated maximal achievable reduction due to these variables in our population. Since lung function parameters result from an expiratory maneuver, a greater effect should indeed be expected from an expiratory CT measure than from an inspiratory measure of emphysema extent.

Our study is of importance as it is the first study to report on the association between expiratory CT data and lung function over time. The study is strengthened by the fact that all scans were obtained according to the same protocol, which excludes interference of scanner differences with the quantitative CT values. Also, our study population was fairly large and population-based; it comprised heavily smoking subjects in a screening setting with mainly absent or mild airflow limitation, instead of severely affected subjects with end-stage disease. Our study is limited by the fact that the generalizability to other less exposed populations, to females subjects and to more severe stages of the disease may be limited.

In conclusion, we showed that expiratory CT air trapping in current and former male heavy smokers without or with mild COPD is independently associated with accelerated decline of FEV_1_/FVC and with reduction of FEV_1_ and FEV_1_/FVC.
